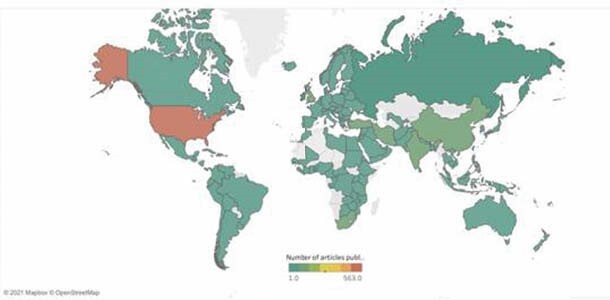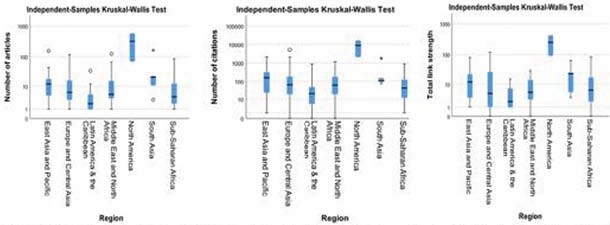# 59 Burns in Low- and Middle-income Countries: A Scientometric Analysis of Peer-reviewed Research

**DOI:** 10.1093/jbcr/irac012.062

**Published:** 2022-03-23

**Authors:** Zachary J Collier, Ulrick S Kanmounye, Priyanka Naidu, Maria Fernanda Tapia, Atenas Bustamante, Daniel Bradley, Chifundo Msokera, John Dutton, William P Magee, Justin Gillenwater

**Affiliations:** Keck School of Medicine, University of Southern California, Los Angeles, California; Operation Smile DRC, Kinshasa, Kinshasa; University of Cape Town, South Africa, Cape Town, Western Cape; Operation Smile Colombia, Bogota, Cundinamarca; Operation Smile Peru, Lima, Lima; Operation Smile UK, London, England; Operation Smile Malawi, Lilongwe, Lilongwe; 6. Rutgers Robert Wood Johnson Medical School, Rutgers University, Princeton, New Jersey; Children’s Hospital Los Angeles, Los Angeles, California; USC/LAC+USC Medical Center, Los Angeles, California

## Abstract

**Introduction:**

Low- and middle-income countries (LMICs) account for 70% of all global burns. Due to this significantly disproportionate burden, it’s critical we identify barriers to burn care and prevention in LMICs. As a result, this study aimed to elucidate trends in LMIC-related burn research to create focused strategies for burn care training, research, and innovation. Accomplishing meaningful change from the study’s findings will be guided by the first 4 steps of Dr. John Kotter’s “8-Step Process for Leading Change” – 1) create urgency for change, 2) build a guiding team, 3) develop a vision and plan, 4) communicate with key stakeholders to obtain buy-in.

**Methods:**

Web of Science’s 7 citation databases were searched through March 2, 2021 using synonyms of “burns” and “low- and middle-income countries.” After screening articles, metadata were uploaded to VOSviewer (Leiden, Netherlands) where citation and network metrics were generated. The Kruskal-Wallis test and linear regression were used for bivariable and multivariable analysis of factors influencing publications, citations, and total link strength (TLS) – the strength of association between a given research article, other articles, and additional institutions.

**Results:**

Bibliometric analysis identified 2,027 articles by 8,602 authors in 692 journals. Two-thirds of journals published a single article (n=453, 65.5%) whereas only 3.6% published ≥10 articles. One-quarter of LMIC burn research was published in ISBI’s Burns (n=417 articles, 20.6%) and ABA’s Journal of Burn Care & Research (n=89 articles, 4.4%). Most authors published < 5 articles (n=8521, 99.1%) but 19 (0.2%) had published ≥10. Authors were affiliated with 2,519 organizations in 132 countries. There was a strong positive correlation between total publications and citations (R=0.87, P< 0.001). In addition, there was a significant difference in the number of publications (P=0.003, 0.07), citations (P=0.005, 0.03), and TLS (P=0.009, 0.008) by geographic and economic categories - North America had the highest while Latin American and the Caribbean had the lowest. The USA (n = 563), India (n = 161), and China (n = 154) published the most articles.

**Conclusions:**

Given the disproportionate representation of high-income countries and authors in the current LMIC burn research landscape, there must be a sense of urgency to develop pathways for facilitating change. Local and regional candidates for mentors and leaders were identified using bibliometric findings. Assembling teams with these individuals and prolific authors using a well-defined vision for change will facilitate sustainable communication and collaboration within LMIC research.